# Evaluation of tear film and the morphological changes of meibomian glands in young Asian soft contact lens wearers and non-wearers

**DOI:** 10.1186/s12886-020-1328-2

**Published:** 2020-03-04

**Authors:** Tianpu Gu, Lu Zhao, Zhuzhu Liu, Shaozhen Zhao, Hong Nian, Ruihua Wei

**Affiliations:** 1 Tianjin Key Laboratory of Retinal Functions and Disease, Tianjin, 300384 China; 2 Eye Institute and School of Optometry, Tianjin, 300384 China; 3 Tianjin Medical University Eye Hospital, Tianjin, 300384 China

**Keywords:** Contact lens, Meibomian glands, Meibomian gland dysfunction, Tear film young Asian

## Abstract

**Background:**

The aim of this study was to explore the differences in terms of tear film and meibomian glands (MGs) between young Asian soft contact lens (CL) wearers and non-wearers.

**Methods:**

A prospective, cross-sectional observational study was conducted using 148 subjects (63 non-wearers, and 85 soft CL wearers who had been wearing CLs for more than 1 year) recruited from a clinic in Tianjin, China. All subjects first responded to an Ocular Surface Disease Index (OSDI) questionnaire and then underwent a standardized dry eye examination, which included measuring tear meniscus height (TMH), non-invasive tear breakup time (NIBUT), and corneal fluorescein staining (CFS). The MGs were evaluated via ImageJ, distorted MG count and the MG dropout were recorded.

**Results:**

Compared to the control group (non-wearers), the CL group recorded higher OSDI and CFS scores, lower TMH and NITBUT values, a larger distorted MG count, and larger MG dropout (all *P* < 0.05). Pearson correlation analysis found a correlation between MG dropout and the duration of CL use (*r* = 0.440, *P* < 0.001), OSDI (*r* = 0.298, *P* = 0.006), and CFS scores (*r* = 0.442, *P* < 0.001).

**Conclusion:**

CL wearers showed higher MG dropout and reduced TMH and NITBUT, which likely contributes to severe CL-related dry eye symptoms. CL use may lead to a higher MG dropout rate, and the extent of the MG dropout presumably influences the tear film status in CL wearers.

## Background

There are an estimated 140 million people worldwide who wear contact lenses (CLs) for refractive error corrections [1]. During the past 20 years in China, there has been an enormous growth in the number of CL wearers, and the number of CL wearers is increasing every year [2].

Dry eye symptoms in CL wearers are more frequent and intense than those in non-wearers [3, 4]. Although lens designs and materials have advanced, a considerable number of soft CL wearers complain of clinically significant signs and symptoms, including foreign body sensations, redness, and CL-related dry eye [5–7]. About 30 to 50% of CL wearers report dry eye symptoms, and dry eye is gradually becoming a public health problem [5, 7, 8]. Moreover, the discomfort or irritation associated with dry eye may lead to intolerance of CLs [9–11]. Such symptoms should be addressed in time to prevent such an occurence [12].

As generally agreed upon, successful CLs wear depends on a stable tear film, including the production and maintenance of the constituents in each of the layers [11]. Several studies have shown that CLs produce a series of biochemical and biophysical changes in the ocular surface, such as in the integrity of the tear film and ocular surface microenvironment [13–15]. Ocular surface health cannot be sustained in the absence of a healthy lipid layer; hence, MG function has attracted the attention of many researchers around the world in recent decades [16]. However, little attention has been paid to the effect of CL on MGs in the Asian population, who tend to have absent or lower lid creases and more fat in the upper eyelid compared to the Caucasian population. Therefore, a better understanding of ethnicity-related variations involving CL discomfort, especially between Asians and non-Asians, must be taken into consideration.

In this study, we use a novel, noncontact meibographic technique to evaluate the morphology of MGs to obtain a more precise calculation of the MG dropout rate in a young Asian population. The purpose of the current study is to explore the differences in the Ocular Surface Disease Index (OSDI), tear meniscus height (TMH), non-invasive tear breakup time (NIBUT), corneal fluorescein staining (CFS) scores, and MGs dropout between soft CL wearers and non-wearers. We also analyze the relationship between MG dropout and the other parameters, including the OSDI, TMH, NIBUT, CFS scores, and the duration of CL use.

## Methods

The cross-sectional observational study was conducted at Tianjin University Eye Hospital (Tianjin, China) in June 2015. This study adhered to the tenets of the Declaration of Helsinki and was approved by the Institutional Ethical Committee Review Board of Tianjin University Eye Hospital. Written informed consent was obtained from all patients before their participation in the study.

All measurements were performed in an identical manner by the same professional operator, who was not aware of the CL wearing status of the subjects before the examinations. All measurements were performed from 9:00 AM to 11:00 AM to exclude the influence of diurnal variations. The ambient conditions of the examination room were kept constant, with the temperature varying from 24 to 28 °C and the relative humidity varying from 40 to 50%. Due to the similar nature of each individual’s two eyes, only the right eye results were analyzed.

A previous study has shown that disruption to the precorneal tear film is short-lived and that the tear film returns mostly to normal within 25 min of CL removal [17]. After the CL was removed and more than 1 h later, the following examinations took place in sequence: TMH, NIBUT, MG dropout, the distorted MG count, and CFS.

### Subjects

A total of 148 consecutive subjects enrolled in the optometry clinic were used in this study (Table 1), and these subjects were divided into the CL group (85 participants, 25.52 ± 3.20 years old, wearing CLs for more than 1 year) and the control group (63 non-wearers, 23.35 ± 3.83 years old).

**Table 1:** Comparison of the age, sex ratio, OSDI, TMH, NITBUT, and CFS scores for participants from both groups

	Age (years)	Sex Ratio (M/F)	OSDI (scale 0–100)	TMH (mm)	NIBUT (s)	CFS scores (scale 0–12)
CL group	25.52 ± 3.20	23/62	17.21 ± 8.67	0.21 ± 0.06	6.95 ± 4.79	2.02 ± 1.48
Control group	23.35 ± 3.83	18/45	11.41 ± 6.00	0.24 ± 0.08	9.26 ± 2.95	1.06 ± 0.88
*P*	0.065	0.622	< 0.001	0.040	< 0.001	< 0.001

*CL* contact lens, *OSDI* the ocular surface disease index, *TMH* tear meniscus height, *NIBUT* noninvasive tear breakup time, *CFS* corneal fluorescein staining, *M/F* male/female

The inclusion criteria for CL group were as follows: more than 18 years old and less than 30 years old; no history of any ocular diseases other than those associated with CL-related changes before wearing CLs; CL replacement based on a monthly or quarterly schedule with storage case replacement every 1 or 3 months; daily (5–7 d/wk., 5–12 h/d) wear, and not using any measures to manage dry eye symptoms, including warm compresses, artificial tear drops, and other medical eye drops. The control subjects were selected with the same age criteria, no contact lens wearing history, and no eye disease other than the dry eye. Exclusion criteria included patients who had active ocular infection or inflammation, recent ocular surgeries, or using medications that might affect the tear film.

### OSDI

The OSDI questionnaire was utilized to quantify ocular discomfort, particularly in terms of measuring symptoms of discomfort related to dry eye. It has been validated and proven to be reliable [18]. The OSDI consists of 12 questions about symptoms of ocular discomfort and dryness. These questions are divided into three parts: vision-related symptoms (questions 1–5), ocular discomfort symptoms (6–9), and environmental stimuli (10–12). Moreover, its total scores range from 0 to 100, which indicate that the ocular surface is normal (0–12 points) or that the individual has mild (13–22 points), moderate (23–32 points), or severe (33–100 points) dry eye disease. All participants were instructed to finish the questionnaire.

### TMH

Using the tear analysis software on the Keratograph 5 M (Oculus, Wetzlar, Germany) [19], TMH was measured at the center of the lower tear meniscus with a graticule in 0.01 mm units. A mean value for the TMH was recorded after three successive measurements of at least 60 s in duration.

### NITBUT

The NITBUT was measured using the Keratograph 5 M with no fluorescein dye, as described in a previous study [20]. After the alignment of the instrument head with the center of the pupil, the participant was asked to blink three consecutive times and then instructed to hold his/her eyes open for as long as possible. The time elapsed between the last blink and the first sign of distortion occurring of the ring pattern was recorded as the NITBUT. The measurement was repeated three times for each eye, and the average was noted.

### Meibography

**Fig. 1:**
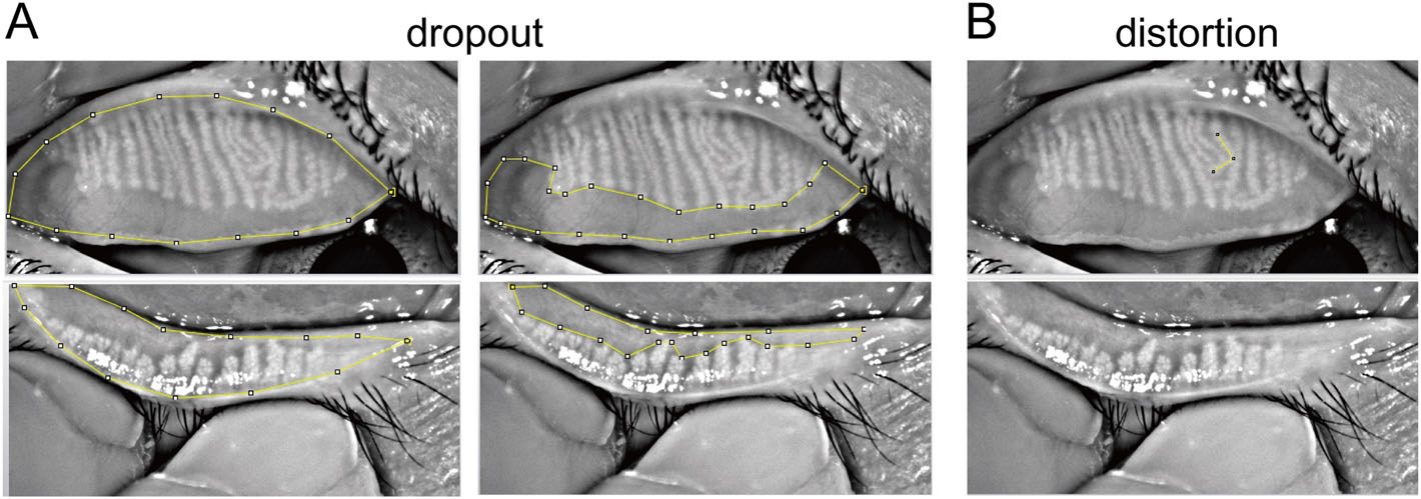
Method to quantity Meibomian gland changes. **a** Meibomian gland dropout. Yellow line in the left panel marks the total area (T), and yellow line in the right panel marks out the area with Meibomian gland dropout (D). The dropout ratio is quantified as the ratio between size of D and T. **b**. Meibomian gland distortion. Yellow line represents Meibomian gland with distortion. The angle between the line segments is greater than 45 degrees

As described in a previous study [21], high-contrast meibography images of MGs were obtained using Keratograph 5 M’s infrared camera system and then analyzed digitally using ImageJ (National Institute of Health; http://imagej.nih.gov/ij). Using a single-handed technique is convenient for the eversion of the upper and lower eyelids when obtaining the meibography images [22]. Using the freehand selection tool in ImageJ, the area of loss was determined by outlining the MG lost area. Then its relation to the total area was calculated and expressed as a percentage, that is, the MG dropout (Fig. 1). The presence of MG duct distortion is established if the morphology in the MG is altered by more than 45 degrees (Fig. 1) [23]. Following this definition, distorted MG count was recorded.

### CFS

To enhance the observation of dry spots, the cornea was stained using single-use disposable fluorescein ophthalmic strips (fluorescein sodium, 1 mg) wetted with nonpreserved buffered saline (sodium chloride, 9 mg/mL). The ocular surface was examined with a biomicroscope under both blue-light illumination and a yellow filter after 2 min of fluorescein sodium staining. CFS was graded from 0 to 12 based on the sum of the four-quadrant scores for the cornea, which were scored individually as 0 (no staining), 1 (mild staining with a few scattered stains), 2 (moderate staining between 1 and 3), and 3 (severe staining with confluent stains or corneal filaments) [24].

### Statistical analysis

Statistical analyses were performed with the SPSS 22.0 statistical package. The data discrepancies between the two groups were determined with the Mann-Whitney U tests for, respectively, age, gender, OSDI, TMH, NITBUT, CFS scores, distorted MG count, and MG dropout. The associations of the total MG dropout with age; gender; the duration of CL use; the OSDI; TMH; NITBUT; CFS scores; and distorted MG count were tested using a univariate model and stepwise multiple linear regression models. The F-test with *p*-values set at 0.05 and 0.1 was used to determine each variable’s enter and exit criteria for the model, respectively (in collinearity diagnostic tests, all Variance Inflation Factors were < 10, indicating no multicollinearity). *P* values lower than 0.05 were considered statistically significant.

## Results

All participants completed all examinations, and none of these subjects experienced any discomfort or pain during the examinations. There were no differences in terms of age (25.52 ± 3.20 vs. 23.35 ± 3.83 years old, *P* = 0.065) and the sex ratio (M/F: 23/62 vs. 18/45, *P* = 0.622) between the CL group and control groups (Table 1). In the CL group, the average duration of CL use was 5.27 ± 4.07 years, ranging from 1 to 18 years.

Compared to the control group (Table 1), the CL group recorded a higher average OSDI (17.21 ± 8.67 vs. 11.41 ± 6.00, *P* < 0.001), a lower average TMH (0.21 ± 0.06 vs. 0.24 ± 0.08 mm, *P* = 0.04), a lower average NITBUT (6.95 ± 4.79 vs. 9.26 ± 2.95 s, *P* < 0.001), and higher average CFS scores (2.02 ± 1.48 vs. 1.06 ± 0.88, *P* < 0.001). Compared to the control group (Table 2), the CL group also recorded a higher average total MG dropout (28.94 ± 12.66 vs. 18.7 ± 8.78%, *P* < 0.001), a higher average MG dropout of the upper eyelid (30.88 ± 13.34 vs. 18.42 ± 8.7%, *P* < 0.001), a higher average MG dropout of the lower eyelid (23.26 ± 17.44 vs. 16.79 ± 11.74%, *P* = 0.017), a larger average total distorted MG count (4.86 ± 3.44 vs. 2.75 ± 1.83, *P* < 0.001), a larger average distorted MG count for the upper eyelid (3.72 ± 3.09 vs. 2.03 ± 1.69, *P* < 0.001), and a larger average total distorted MG count for the lower eyelid (1.14 ± 0.97 vs. 0.71 ± 0.63, *P* = 0.006).

**Table 2:** Comparison of the MG dropout and distorted MG count for participants from both groups

	MG dropout (%)			Distorted MG count		
	Total	Upper eyelid	Lower eyelid	Total	Upper eyelid	Lower eyelid
CL group	28.94 ± 12.66	30.88 ± 13.34	23.26 ± 17.44	4.86 ± 3.44	3.72 ± 3.09	1.14 ± 0.97
Control group	18.7 ± 8.78	18.42 ± 8.7	16.79 ± 11.74	2.75 ± 1.83	2.03 ± 1.69	0.71 ± 0.63
*P*	< 0.001	< 0.001	0.017	< 0.001	< 0.001	0.006

*CL* contact lens, *MGs* meibomian glands

**Fig. 2:**
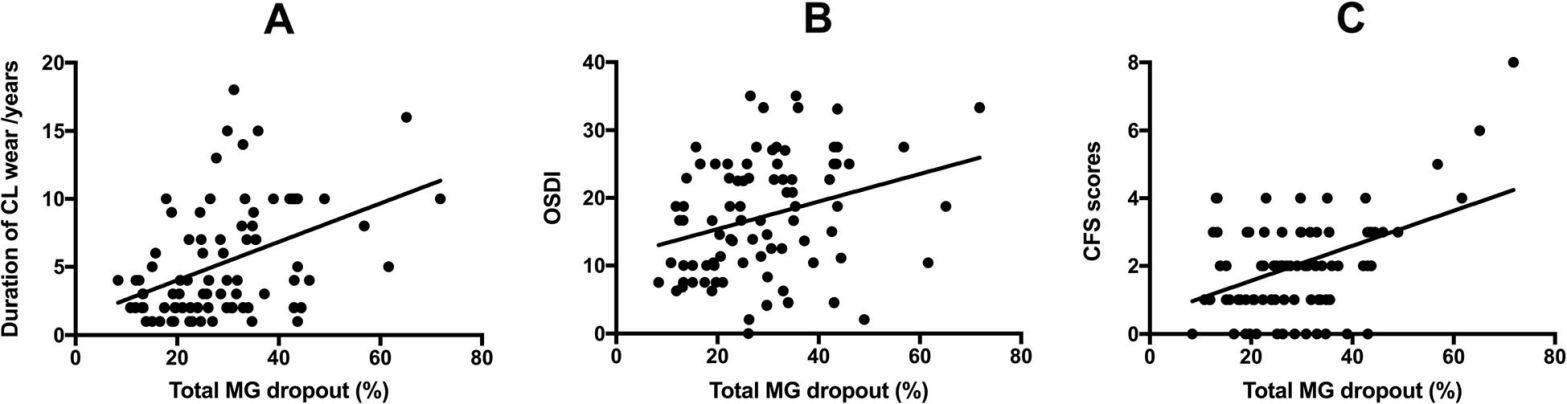
Scatterplot of the total MG dropout by duration of CL use (β = 0.014, *P* < 0.001, **a**), OSDI (β = 0.004, *P* = 0.006, **b**), and CFS scores (β = 0.038, *P* < 0.001, **c**) with the best fit line in soft CL wearers

In the univariate analysis (Table 3), a larger total MG dropout was associated with the older age of the subject (*P* < 0.001), larger OSDI (*P* = 0.006, Fig. 2b), larger CFS scores (*P* < 0.001, Fig. 2c), and longer duration of CL use (*P* < 0.001, Fig. 2a). In the stepwise multivariate analyses, three factors were associated with the total MG dropout (*R*^2^ = 0.356, *P* < 0.001, Table 3): OSDI (β = 0.003), CFS (β = 0.031), and the duration of CL use (β = 0.010).

**Table 3:** Linear regression analyses of the associations of the total MG dropout with age, gender, OSDI, TMH, NIBUT, CFS scores, the number of distort MGs, and the duration of CL use

	Univariate model		Multivariate model	
	beta (95% CI)	*P*	beta (95% CI)	*P*
Age (years)	0.016 (0.008 to 0.024)	< 0.001	–	–
Gender (male)	−0.037 (−0.099 to 0.024)	0.229	–	–
OSDI	0.004 (0.001 to 0.007)	0.006	0.003 (0 to 0.005)	0.043
TMH (mm)	0.187 (−0.276 to 0.065)	0.424	–	–
NIBUT (s)	−0.003 (− 0.009 to 0.003)	0.311	–	–
CFS scores	0.038 (0.021 to 0.055)	< 0.001	0.031 (0.016 to 0.047)	< 0.001
Number of distort MGs	0.003 (−0.005 to 0.011)	0.477	–	–
Duration of CL use (years)	0.014 (0.008 to 0.020)	< 0.001	0.010 (0.004 to 0.016)	0.001

*CL* contact lens, *OSDI* the ocular surface disease index, *TMH* tear meniscus height, *NIBUT* noninvasive tear breakup time, *CFS* corneal fluorescein staining, *MGs* meibomian glands

**Fig. 3:**
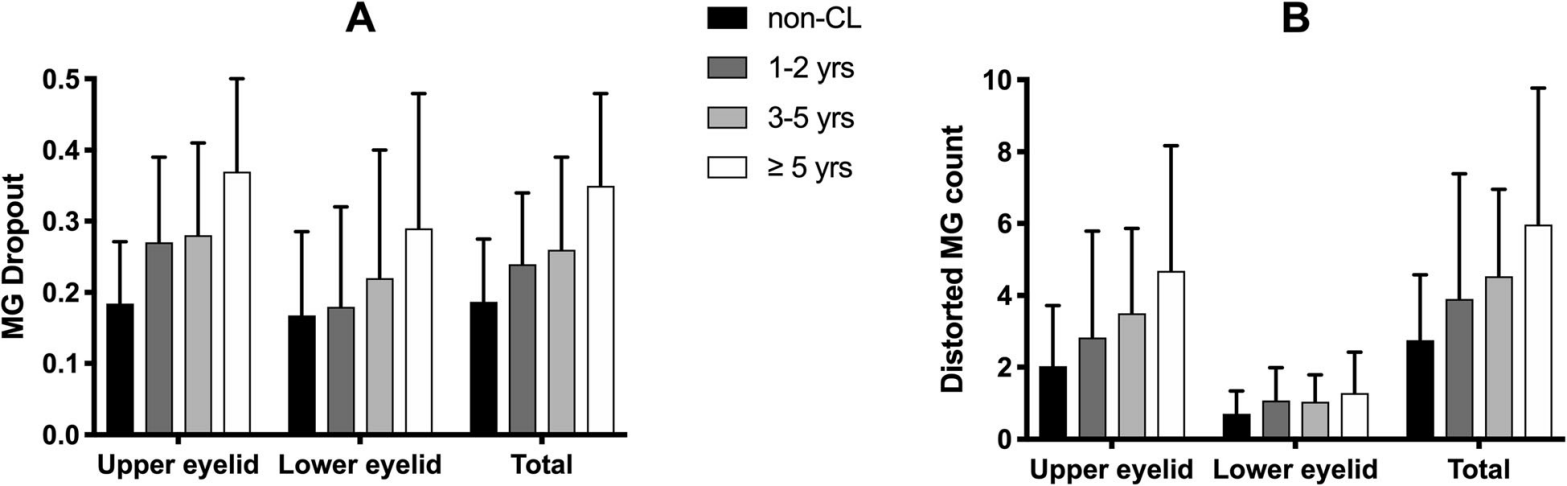
Comparison of the MG Dropout and Distort MG count for Participants from Both Groups (the CL group were divided into the three small groups by the duration of CL wear: 1–2 years, 3–5 years, and over 5 years)

In Fig. 3, the CL group is divided into three small groups according to the duration of CL use (1–2 years, 3–5 years, and over 5 years) for a more intuitive look at the changes in MG dropout and distorted MG count.

## Discussion

A controversy exists as to whether CL use has an impact on the health of the ocular surface. Chong et al. [25] reported that there was no significant change in the function of the tear film after 7 days of CL use, and Sapkota et al. [7] reported that CL-related discomfort was not associated with the duration of CL use. However, Dogan et al. [26] found that patients with CL-related discomfort usually had a longer CL use history than those without symptoms. Chalmers et al. [27] showed that CL wearers in North America reported longer hours of use with significant aggravation of symptoms of discomfort compared to CL users in the United Kingdom. These previous studies [25–27] regarding CL-related discomfort were all short-term investigations, with the exception of Sapkota’s [7]. In the present study, the CL wearers recorded higher OSDI, lower TMH, lower NITBUT, and higher CFS scores. These findings suggest that CL use gives rise to the increasing severity of CL-related discomfort. Our findings of worsening tear film and quicker evaporation rates in CL wearers agree with the previous studies [10, 17, 28].

The MGs are large sebaceous glands located in the eyelid. The lipid substances these glands secrete form the lipid layer of the tear film, which acts to prevent tear vaporization and promotes tear film stability. Therefore, disrupting the normal activities of the MGs will alter normal tear physiology through the thinning and breakup of the tear film, interruption of tear film reformation, and disruption of the lipid layer, which results in increased tear film evaporation [29, 30]. Although MG dropout is the most common cause of evaporative dry eye [31], little attention has been paid to a potential role of MG morphologic changes in CL wearers’ dry eye symptoms.

Some previous studies have reported that long-term CL use leads to an increase in the MG dropout [32–35]; however, this effect has not been observed in other studies [36–38]. One constraining factor for the application of these findings to other populations is race. The only subjects in Arita’s study were Asians, who have an absent or lower crease and more fat in the upper eyelid than Caucasians [39]. The subjects of Other studies were Caucasians [32, 34, 35, 40]. Mechanical trauma from the CLs was determined to cause duct blockage in the MGs [33], and is proportional to the tightness of the eyelid. The results of this study indicate that CL wearers have a higher degree of MG dropout and a larger distorted MG count than non-wearers (Fig. 3). This suggests that CL use is associated with reduced MG morphology, which is consistent with previous reports [33, 35]. The study by Arita et al. [33] investigated MG dropout only, and did not assess distorted MG count. Furthermore, since our study uses a contemporary infrared system, the calculation of MG dropout is more precise.

Aging increases the severity of MG changes in normal individuals [41], which may account for the lower MG dropout rate in the control group. However, Den et al. showed that only a few patients aged less than 50 years had notable abnormalities in the MG [42], but studies also suggest that CL wear accelerates age-related changes in the MGs [32–35]. Compared with other studies, we used a younger population to explore the effects of CL on the tear film and MG morphology, as this population is known to be more likely to wear CL for a long time. The average age difference between the study and control group was 2.17 years in this study. However, we think the two-year age gap between the 18–30 age range would not make a statistically significant difference based on the above reason. We found that age was significantly associated with the total MG dropout in the univariate linear regression analysis but not in the stepwise multiple linear regression analysis. A significant positive relationship was found between MG dropout and the duration of CL use in both the univariate and multiple linear regression analyses, which is in agreement with previous studies [33]. However, Alghamdi et al. [35] reported that alterations to MG morphology and function accompany CL wear during the first 2 years of wear, with no further deterioration thereafter. In the early stages of CL use, MG dropout increased significantly, but no further deterioration was found after that [35].

We found a significant positive relationship between MG dropout and OSDI, as well as CFS scores. These results suggest that MG dropout may affect CL-related dryness and maybe one of the potential mechanisms underlying CL-related dry eye. However, Nichols et al. [28] found that the extent of MG dropout was not related to dry eye status in CL wearers. The reason may be that only the MGs in the lower eyelid were analyzed in their study. Both upper and lower eyelids were evaluated in our study, and the total MG dropout was used for the statistical analyses. Similar to the results found in a previous study [33], we found that MG dropout in the upper eyelid (30.88 ± 13.34%) for CL wearers was significantly higher than that of the lower eyelid (23.26 ± 17.44%), and the MG dropout in upper eyelid was closer to the total than that in the lower eyelid. This finding suggests that using the total MG dropout for both eyelids in analyses may be more reliable. This evidence also supports that chronic CL-mediated irritation through conjunctiva is a major contributor to MG changes in CL wearers [33, 43] and also that it has a more considerable influence on the MGs of the upper eyelid, especially during blinking.

Complete and functional MGs are essential for normal tear film, particularly in the lipid layer. A healthy lipid layer is integral to tear quality as it provides the tear film with structural integrity [44]. Therefore, damage to any component of an MG alters the tear quality, potentially leading to dry eye [16]. In addition, a build-up of meibum in an MG causes ductal dilation, cystic degeneration, loss of the meibocytes, and eventual gland atrophy [16]. Due to the higher degree of MG dropout found in CL wearers, which is supported by the previous [28, 33, 45] studies, the reduction in meibum volume and quality when secreted onto the ocular surface lead to alterations such as increased evaporation due to the thinning of the lipid layer [16, 31, 46, 47]. Thus, MG dysfunction accompanied by MG dropout presumably plays an important role in the dry eye in CL wearers [48]. However, no significant relationship between MG dropout and TMH (*r* = 0.088, *P* = 0.424) and MG dropout and NIBUT (*r* = − 0.111, *P* = 0.311) was found in this study.

There are several limitations worth noting. First, a large number of studies have shown that the lens material, surface properties, and parameters play a role in CL-related discomfort and that changes in the material, design, and fitting characteristics of CLs may reduce CL discontinuation [13, 49–51]. However, this study was limited to the study of a soft CL group versus a non-CL group and did not include additional groups with different CL designs. Second, the refractive error was not adjusted between the CL and control groups, although the refractive error involved has shown no correlation with CL-related dry eye. Third, the current study did not evaluate the lid margin and MG function. In future studies, a consideration of such as the lipid layer, changes in MG orifices, line of Marx, tarsal plats, tear quantity, tear evaporation, and osmolarity should be considered.

## Conclusions

In conclusion, CL wearers showed higher MG dropout and reduced TMH and NITBUT, which likely contributes to CL-related dry eye symptoms. CL use may lead to a higher MG dropout, and the extent of MG dropout presumably influences the tear film status in CL wearers.

## Data Availability

The datasets used and/or analyzed during the current study available from the corresponding author on reasonable request.

## Abbreviations

CFS: Corneal fluorescein staining
CL: Contact lens
MGs: Meibomian glands
NIBUT: Noninvasive tear breakup time
OSDI: The ocular surface disease index
TMH: Tear meniscus height
